# Subacute Sclerosing Panencephalitis: Impact on Public Health, Current Insights, and Future Perspectives

**DOI:** 10.1002/brb3.70292

**Published:** 2025-02-09

**Authors:** Zainab Mubbashir, Zoaib Habib Tharwani, Tilyan Kambar, Sadia Munawar, Ozem Raphael, Iman Siddiqui, Syeda Ayesha Nadeem, Ayesha Amir, Amina Ahmed, Muhammad Daim Bin Zafar, Muhammad Umair Anjum, Muhammad Hasanain, Abdullah Malikzai

**Affiliations:** ^1^ Department of Pharmacology Karachi University Karachi Pakistan; ^2^ Faculty of Medicine, Dow Medical College Dow University of Health Sciences Karachi Pakistan; ^3^ Faculty of Medicine Ziauddin Medical College Karachi Pakistan; ^4^ Faculty of Medicine Jinnah Sindh Medical College Karachi Pakistan; ^5^ Faculty of Medicine Kabul University of Medical Sciences Kabul Afghanistan

**Keywords:** antiviral agents, measles resurgence, neurological disorder, SSPE

## Abstract

**Background:**

Subacute Sclerosing Panencephalitis (SSPE) is a rare complication of the measles infection. SSPE is a chronic disease of the central nervous system (CNS) that causes encephalitis, leading to the demyelination of neurons in the brain. It affects the brain in 9 months or less and hence subacute, causing encephalitis and lesions in the entire brain, so the term panencephalitis is used.

**Methods:**

A comprehensive literature search was conducted using the databases PubMed and Google Scholar starting in April 2024, and all relevant articles were extracted for this review.

**Results:**

A recent surge in SSPE cases in developed countries has been reported. This has been attributed to reduced vaccination, aggravated by misinformation and a decline in immunization after the COVID‐19 pandemic. SSPE is a progressive and relatively rare neurological complication of measles, which almost always results in a vegetative state followed by death. It typically presents 10 years following exposure to measles. Manifestations of SSPE are divided into four stages that range from general personality changes to coma. Complications include ocular pathology and eventual blindness, as well as psychiatric illnesses. Treatment options for SSPE include symptomatic control with antiepileptic drugs, interferon combined treatment, vitamin A, ribavirin, and a ketogenic diet for disease modification.

**Conclusion:**

The only prevention for SPPE is through vaccination. Several collaborative efforts have been made with WHO to improve surveillance and increase vaccination, but still many challenges prevail. Better prevention strategies and improved treatment outcomes can only be achieved by enhancing healthcare access, improving public awareness, analyzing community‐based data, and studying the genetic and molecular associations of measles and SSPE.

## Graphical abstract

Subacute sclerosing panencephalitis (SSPE) is a severe complication of measles that demands global attention. The only way to eradicate measles and prevent its complications is through widespread immunization and increased public awareness.

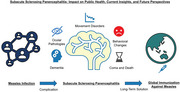

## 1 Introduction

Subacute sclerosing panencephalitis (SSPE) is a rare and invariably fatal characterized by progressive inflammation and degeneration of the brain tissue secondary to infection by the measles virus (MeV) (World Health Organization 2024; Gutierrez and Singer 2010). First identified by Dawson and L Van Bogaert (Lebon et al. 2021), the condition was named based on its distinctive features, including relatively rapid onset (subacute), discernible pathological changes observed (sclerosis), and the extensive involvement of the entire brain (panencephalitis). SSPE occurs once per 5000 measles cases (Com 2022), typically manifesting 7–10 years after an initial measles infection.

Despite efforts of the World Health Organization (WHO) and Centers for Disease Control and Prevention (CDC), there has been an 18% increase in estimated measles cases and a 43% increase in estimated measles deaths in 2022 compared with 2021, with large or disruptive outbreaks being reported in 37 countries due to its highly infectious nature. SSPE continues to pose a significant threat to public health, particularly in regions with suboptimal vaccine coverage (Minta et al. 2023; Lee et al. 2023; D. Garg and Sharma 2023). Furthermore, the limited treatment options available for SSPE underscore the urgent need for research to identify innovative therapeutic approaches.

The scarcity of literature on SSPE, its high mortality rate, and the lack of validation in diagnostic criteria for atypical presentations highlight the importance of investigating the limited number of cases (Gutierrez and Singer 2010; Lebon et al. 2021; D. Garg and Sharma 2023). By analyzing clinical data and epidemiological trends, this study seeks to provide insights into SSPE's pathogenesis, clinical course, and management strategies. Moreover, we discuss the public health aspect of SSPE to understand the preventative strategies better, highlight the current efforts, and provide recommendations to reduce the prevalence.

## 2 Methodology

This narrative review discusses the relevant literature, including updated studies concerning the SSPE and its public health impact. The literature search for studies was performed from inception to April 20, 2024, using the databases PubMed and Google Scholar. The search terms included “subacute sclerosing panencephalitis,” “measles,” “public health,” and “epidemiology.” We included articles in the English language that were open‐access and full text. Moreover, external sources such as the WHO, CDC, NHS, etc., were also used for relevant information. Abstract‐only articles, commentaries, letters to the editor, and articles in languages other than English were excluded from this review.

## 3 Pathogenesis and Clinical Features

**FIGURE 1:**
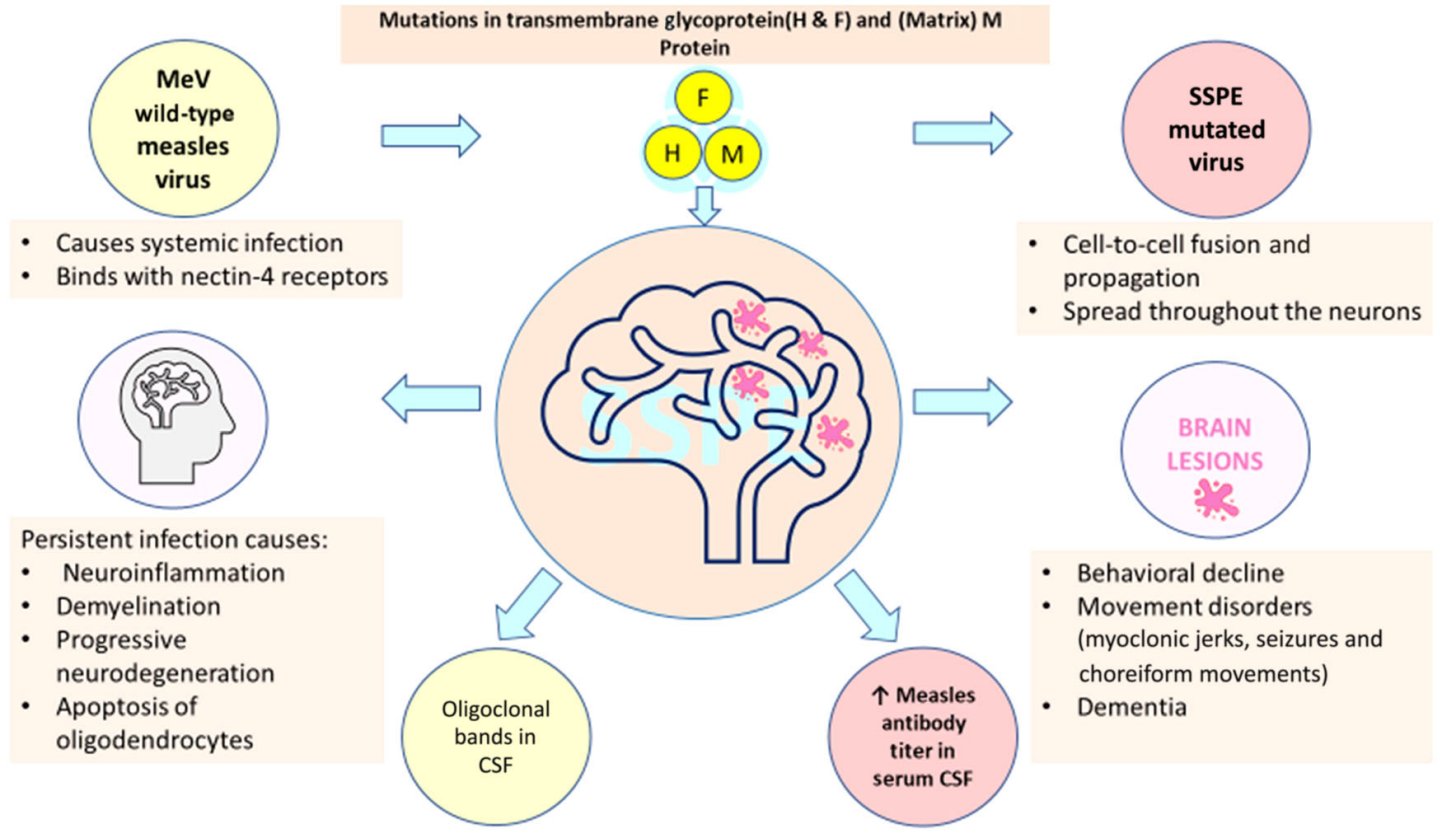
Pathogenesis of subacute sclerosing panencephalitis.

MeV is a single‐stranded negative‐sense RNA virus of the Paramyxoviridae family that acts by infecting both the B and T lymphocytes. Infection of the B‐cells forms syncytial Warthin–Finkeldey in the upper gastrointestinal tract. The virion migrates in the body and attaches to the epithelial cell surface by binding to the nectin‐4 receptors, causing systemic infections (Figure 1) (Upadhyayula et al. 2017). This virus has an R0 of 12–18, meaning that an infected person can transmit the infection to 12–18 healthy individuals; this rate persists even with the availability of vaccines (Ferren and Mathieu 2019). MeV was previously thought to have no gender predisposition; however, a study reported a greater frequency among males than females, with a male‐to‐female ratio of 3:1 (Cece et al. 2011).

The clinical features of measles commonly reported are fever, cough, coryza, maculopapular rash within 3 days, conjunctivitis, and Koplik spots. The complications in infants are more attenuated, with bronchopneumonia being the most significant (Sindhu et al. 2019). Measles, blindness keratitis, corneal ulceration, and corneal scarring are associated with vitamin A deficiency (Semba and Bloem 2004). One of the studies reported that around 10%–50% of patients usually suffer from SSPE‐associated ocular pathologies, including macular and peri‐macular chorioretinitis, papilledema, and papillitis (Figure 1). More serious complications include optic atrophy, cortical blindness, and Anton's syndrome (R. K. Garg 2002). Following a measles infection within the Central Nervous System (CNS), the virus establishes a latent presence in the brain, evading immune surveillance mechanisms (Gutierrez and Singer 2010). Factors such as impaired immune responses and genetic predispositions contribute to viral reactivation, leading to progressive neuroinflammation, demyelination, and neuronal degeneration (Lebon et al. 2021; Ferren and Mathieu 2019) (Figure 1). Precise mechanisms governing viral latency, spread, and reactivation in SSPE remain areas of active investigation.

SSPE is a late complication of measles, categorized as a progressive neurodegenerative disorder. It usually occurs around 10 years after exposure to MeV with a latency period varying between 1 month and 27 years (Samia et al. 2022). A shorter latency has been observed in familial SSPE cases and children affected at a younger age (< 2 years), with incidence rates of 18/100,000 among those under 5 and 1.1/100,000 in those affected after age 5 (Sharma et al. 2008; Gutierrez, Issacson, and Koppel 2010). The wild‐type MeV is not found in the brains of SSPE patients because the MeV genome undergoes mutation. The mutation occurs in the viral transcripts, causing translational arrest, there is defective viral replication that allows MeV to evade host immune surveillance and survive in the CNS. In the virus particles recovered from the brains of SSPE patients, it is evident that the MeV genome undergoes clustered mutations. This causes uracil to cystine transitions in the M gene, causing hypermutation, and this mutated MeV keeps replicating within the brain parenchyma. In SSPE patients there is a high measles antibody titer in the serum and CSF for all the measles proteins except the M protein. Several case reports on SSPE have also mentioned this characteristic elevation of antimeasles IgG and IgM antibodies in the serum and the presence of oligoclonal bands in the CSF (Karthik et al. 2024; R. K. Garg, Pandey, et al. 2023). According to the Dyken criteria, oligoclonal bands can be used as a measure for the diagnosis of SSPE; the absence of oligoclonal bands in the CSF eliminates SSPE as a possible diagnosis (Rinawati and Kumalawati 2022). The signaling lymphocyte activation molecule (SLAM) and nectin‐4 receptors are the cell receptors that allow the MeV to enter the cell, but the CNS lacks SLAM and nectin‐4 receptors; therefore, wild‐type MeV cannot enter the brain. There are alternate routes of entry, such as infectious monocytes in the peripheral blood that can carry the virus to the brain, viral particles can be released into the brain parenchyma postviral replication in the endothelial cells, or via the olfactory bulb (Liebert 1997; Patterson 2020; R. K. Garg et al. 2019).

1. The MeV first enters into the brain during the exanthematous acute phase of the measles infection
2. Then the virus infects the neurons that lack the receptors for MeV
3. The transfer of transmembrane and cytoplasmic protein between cells is facilitated through the nectin‐mediated cytoplasmic transfer via the nectin adhesive interface, enabling MeV to spread from epithelial cells to primary neurons
4. MeV evades immune recognition by undergoing multiple mutations within the neuron
5. As mentioned above, the M gene is the highly mutated gene in almost all the SSPE cases, which promotes the escape of MeV from neutralizing antibodies
6. The persistent infection in the CNS is established through intracellular noncytopathic reproduction of MeV, and the host neurons are not destroyed during this process
7. The cell‐to‐cell fusion transfer and transneuronal viral spread take place due to the hyperfusogenicity of F protein, and neurokinin‐1 or cell molecules 1 and 2 often allow MeV to induce neuronal fusion
8. For the transition from persistent to reproductive infection, mutations of the F proteins are essential. When the clinical symptoms of SSPE occur (e.g., decreased school performance, seizures, and behavioral changes), the SSPE virus is widely spread throughout the CNS
9. The brain's inflammatory response to the persistent SSPE infection leads to widespread tissue damage and cerebral atrophy; florid panencephalitis is a characteristic of SSPE (Sakamoto et al. 2023)

The persistent infection due to the mutated MeV leads to mononuclear inflammation, demyelination in the brain, and cell death of neurons and oligodendrocytes due to apoptosis. The activation of CD4^+^ and CD8^+^ T cells, coupled with monocytes and antibody‐screening B lymphocytes, is responsible for the mononuclear inflammatory response in the brain (R. K. Garg et al. 2019).

The clinical manifestations of SSPE are grouped into four stages. Stage 1 is associated with general personality changes, followed by stage 2, where frequent and repetitive myoclonic jerks, seizures, and dementia occur. Distinctive weaknesses such as speech disorders, loss of vision, and limb weakness are also manifestations of stage 2. Stage 3 includes extrapyramidal symptoms (EPS) and progressive unresponsiveness, and lastly, stage 4 involves coma, vegetative state, and akinetic mutism (Mekki et al. 2019). Hemi‐parkinsonism, chorea, dystonia, and certain other isolated EPS‐associated are marked as atypical presentations of SSPE that make diagnosis challenging (Jafri et al. 2018). The most common psychiatric disorders associated with SSPE included schizophrenia, catatonia, and other atypical psychotic illnesses, reported among 63% of the patients (R. K. Garg et al. 2024). A recent study comprising 21 cases outlines the obstetric outcomes of SSPE, indicating that visual loss along with progressive encephalopathy is the most significant clinical feature observed in pregnant women, followed by headaches, psychiatric and focal deficits, and seizures. The maternal outcomes of the study highlighted that most of the women died after delivery with most fetuses being alive but delivered preterm (R. K. Garg, Paliwal, et al. 2023).

Jafri et al. have divided Dyken's criteria into six subdivisions, including two majors and four minors. The major criteria entail (1) elevated antimeasles antibody titers in the cerebrospinal fluid (CSF), the ratio being ≥ 1:4 or serum level ≥ 1:256; and (2) clinical history of typical and atypical presentations. Minor criteria include (1) Radermecker complexes (periodic slow waves with fixed intervals on electroencephalographic (EEG) including periodic generalized bilaterally synchronous and symmetrical slow waves recurring at 5–15 s); (2) greater than 20% of the total CSF protein comprising of CSF globulins; (3) brain histopathology shows necrotizing leukoencephalitis, diffuse demyelination, inflammatory meningeal changes, viral inclusion bodies in oligodendrocytes and astrocytes, with neuronal loss and astrogliosis; and (4) molecular diagnostic testing identifying wild‐type of MeV. To ensure the diagnosis, two major and one minor criterion are required (Jafri et al. 2018).

Neuroimaging also helps build a differential diagnosis of SSPE but is usually not diagnostic. The MRI findings are generally supportive of diagnosis, with abnormal MRI findings being observed mostly in the later stage of the disease when a significant area of the brain parenchyma is already affected (Mekki et al. 2019). A study identified the pathologies on MRI, which entails white matter hypersensitivity along with cerebral atrophy associated with sulci dilation and gyri atrophy along with dilation of ventricles in the frontal, parieto‐occipital, and temporal regions (Keerthiraj et al. 2024).

A recent study has also shown that TAR DNA‐binding protein 24 (TDP‐43) pathology is associated with SSPE. For this study. Sixteen autopsied brains of SSPE patients were examined, and 31% of cases had TDP‐43 inclusions. TDP‐43 is a nuclear protein associated with transcriptional regulation and alternative splicing; its cytoplasmic aggregation is a pathological hallmark for many neurodegenerative diseases and viral infections. These TDP‐43 inclusions in SSPE are linked to longer disease duration (> 4 years) along with the occurrence of tau pathology. All of these cases also had tau‐positive neurofibrillary tangles (Acewicz et al. 2024).

### 3.1 Molecular Hallmarks of SSPE MeV Versus Standard MeV

MeV comprises two nonstructural proteins and six structural proteins. MeV carries two transmembrane glycoproteins, which are the hemagglutinin (H) and fusion (F). H protein is comprised of two domains, a head that binds with the receptors and the stalk domain that is responsible for delivering a fusion signal to the F protein. Both H and F proteins form a fusion complex that allows the virus to enter into the host cell. The ribonucleoprotein complex is comprised of nucleo‐(N), phospho‐(P), and large‐(L) proteins that are essential for the protection of RNA, its replication, and protection (Schmitz et al. 2024).

The SSPE virus is a highly mutated version of MeV. Mutations in the F protein make the protein hyperfusogenic, facilitating fusion despite the lack of MeV receptors in the neurons. This gives rise to progressive cell‐to‐cell spread of mutated MeV genome throughout the CNS. Hyperfusogenicity has been linked to altered stability of the F protein; therefore, the pre‐ to postfusion conformational changes take place quicker in these F proteins than in the normal “stable” F proteins. Hypermutation of the M gene is also a hallmark characteristic of SSPE. Cell‐to‐cell spread is the only alternative for the virus to spread throughout the CNS, meanwhile ablating the production of cell‐free viruses. Immunomodulatory enzymes, adenosine deaminase acting on RNA (ADAR), are often responsible for these mutations (Takemoto et al. 2022). The F protein has mutations in one specific amino acid in the L454W position. It increases the F protein's fusion ability with other cells and reduces its thermal stability. It is also known that this MeV mutation in the F protein (L454W) is sufficient to cause CNS infection in humans. However, mutation does not only occur in the F protein; L and P proteins also take part in mutations that cause cell‐to‐cell fusion. In the nonmutated MeV, the (matrix) M protein produces infectious viral particles. In the SSPE virus, a bundle of mutations occurs around the M protein, and this damaged M protein inhibits the release of viral particles, which then enhances cell–cell propagation. M protein indirectly enhances the neurovirulence of SSPE and helps establish a persistent infection in the CNS (Satoh et al. 2021). Identifying such mutations is important to gain insight into the pathogenesis of SSPE progression and is valuable for guiding future research (Mathieu et al. 2021; Sakamoto et al. 2022).

It has also been determined that cell adhesion molecules (CADM1 & CADM2) are the host factors that facilitate MeV to cause membrane fusion and for the virus to spread between neurons without known receptors. CADM1 and CADM2 have a cis‐acting binding with the MeV attachment protein that triggers membrane fusion of the neuropathogenic MeV mediated by hyperfusogenic F proteins (Shirogane et al. 2021). Furthermore, only short‐stalk isoforms of CADM1 and CADM2, primarily expressed in the brain, trigger this membrane fusion. These isoforms interact in cis with the H protein even if it lacks a head domain and can cause membrane fusion seemingly through its stalk domain (Takemoto et al. 2022).

## 4 Epidemiology

The global prevalence of SSPE is approximately 4–11 cases per 100,000 cases of measles worldwide (M. Garg et al. 2022). Measles occurring during infancy, particularly before 12 months of age, increases the likelihood of SSPE to 18 cases per 100,000 children (Papetti et al. 2022). A local population survey in Germany showed that children under 5 who had measles had a 1 in 1700 to 1 in 3300 chance of developing SSPE. Children under the age of 3 faced a nearly twofold increased risk. In California, children under 5 had a 1 in 1367 risk, and those under the age of 1 had a 1 in 607 risk of postmeasles SSPE (Mekki et al. 2019).

The widespread implementation of measles vaccination programs has considerably decreased SSPE incidence in several countries, but it is still significant in emerging economies, especially in Asia and Africa (Hiremath et al. 2022; Rocke and Belyayeva 2023). However, the recent surge in SSPE cases is due to reluctance towards vaccination, poor patient education, and fewer initiatives for early immunization (Hiremath et al. 2022). Additionally, the COVID‐19 pandemic has also impacted SSPE vaccination efforts (Papetti et al. 2022), causing disruptions in routine immunization programs and potentially leading to delays in monitoring and diagnostics of SSPE cases.

## 5 Risk Factors and Preventive Measures

MeV is an airborne pathogen transmitted by inhalation of droplets and through direct contact with infected individuals; the virus does not survive for a long time on the fomites as it is inactivated by heat and UV radiations (Rota et al. 2016). Several risk factors increase young children's susceptibility to contracting measles and developing SSPE. These include undernutrition, poor socioeconomic conditions, limited parental education, lack of measles vaccination, having more siblings, being born later in the birth order, and increased likelihood of exposure to measles before the age of 5 (Mekki et al. 2019; Rota et al. 2016).

Prevention of SSPE primarily involves preventing measles. This can be achieved through vaccination. The measles vaccine is relatively cheap and easily available; still, a huge part of the population remains unvaccinated for several reasons, the most common being a lack of awareness among people living in rural areas and the lack of campaigns (Shirogane et al. 2021). Infants born to unvaccinated mothers are at higher risk of contracting measles right after birth and therefore have the highest risk of developing SSPE. Antimeasles antibodies transmitted through the placenta can temporarily protect infants from measles; however, the sustainability varies (Pittet and Posfay‐Barbe 2020). To prevent outbreaks and transmission to infants too young to be vaccinated, more than 95% of the population should be immune to measles (Hens et al. 2015). The WHO advises the administration of the initial dose of the measles‐containing vaccine (MCV) at 9 months where measles is prevalent and at 12–15 months in the nonendemic regions (Coughlin et al. 2017).

## 6 Public Health Implications of SSPE and Measles

As per the WHO goals, the UK aims to reach 95% coverage for childhood immunizations by age five. However, vaccination rates in the 2022/23 period did not meet this target. Vaccination rates in 2022–23 declined across 12 out of 14 metrics, with none reaching the 95% mark. The most recent vaccination to surpass the 95% threshold was the 5‐in‐1 vaccine at age 5 years, with a 95.2% coverage rate in 2020–21. MMR1 and MMR2 coverage fell to 92.5% and 84.5%, respectively, by age 5 years. Previously the global yearly occurrence of measles declined from 145 to 25 cases per million between 2000 and 2017 (Dabbagh et al. 2018). However, almost 150,000 measles cases were reported globally in 2020, according to the WHO, with Africa and Asia accounting for the majority. In Africa alone, the number of cases exceeded 115,000 in 2020. There is a recent surge in measles incidence in the United Kingdom that requires attention in order to meet their preset goal (NHS England Digital). This surge indicates the presence of factors hindering vaccination efforts, leading to reduced immunization and increased susceptibility to measles and thus SSPE (Memon et al. 2021).

Challenges of vaccination coverage are a major issue, as is the inadequate vaccination of eligible individuals despite the availability of supplemental immunization programs and routine immunization services. In South African countries, the problem is made worse by reluctance toward measles vaccination campaigns, as well as by nomadic lifestyles and some apostolic religious congregations who are unwilling to vaccinate. Furthermore, parental attitudes and knowledge influence a child's immunization schedule (Mekki et al. 2019). One of the reasons behind the resurgence of measles outbreaks in countries offering vaccination services is the growing number of people opting to delay or decline vaccination for themselves or their children. This trend, termed vaccine hesitancy by the Strategic Advisory Group of Experts (SAGE), contributes immensely to the problem (NHS England Digital). Ensuring the safety of immunizations is a critical global priority, and it has remained a great challenge, involving three key aspects: vaccine safety, safe administration, and program surveillance. Unsafe injection practices in the developing world pose significant health risks, contributing to the spread of blood‐borne diseases like hepatitis B and C, as well as HIV, which may further influence vaccine hesitancy. This highlights the importance of prioritizing injection safety, especially in immunization programs (PRB).

The Global Vaccine Action Plan (GVAP) 2011–2020, an extensive framework to advocate for widespread access to immunization, introduced the global vaccine availability indicator as a critical assessment metric (Lee et al. 2023). The drive to improve the existing vaccine delivery system arises from various concerns, including the need to sustain or reduce the cold chain requirements, ensure injection safety, manage contaminated waste disposal, and address the shortage of medically qualified personnel available to administer vaccinations (Coughlin et al. 2017). Efforts to improve vaccine surveillance have also been made by the Global Measles and Rubella Laboratory Network (GMRLN) in collaboration with the WHO, which conducts surveillance based on individual cases, employing standardized methodologies (Mulders et al. 2016).

The Immunization Agenda 2030 (IA2030) program by WHO outlines a global vision and strategy for vaccines and immunization from 2021 to 2030, developed with contributions from numerous countries and organizations worldwide to address existing and emerging challenges posed by infectious diseases like Ebola and COVID‐19. IA2030 strives to cater to the interests of every nation and aims to mobilize stakeholders towards ensuring universal access to vaccines for improved health and well‐being. The strategy is implemented through regional and national frameworks to ensure accountability and ownership. However, substantial investments and program improvements are required, particularly in low‐ and middle‐income countries, to meet the 2030 targets (World Health Organization). These global surges in measles also underscore the value of healthcare practitioners’ awareness of the potential rise in SSPE cases and keeping that as a possible diagnosis for pediatric patients presenting with symptoms of neurological decline. This also raises the question of whether we should start earlier scheduling of measles vaccination to prevent SSPE. A recent analysis has also validated the efficacy of the vaccine at 6–9 months to prevent measles exposure during infancy (Lam et al. 2021).

Enhancing healthcare access for SSPE patients and measles‐afflicted individuals, particularly in resource‐constrained regions, requires a comprehensive approach. Automating vaccination delivery, handling, stock management, storage, and monitoring systems is essential for ensuring vaccine availability in a viable form (Samia et al. 2022). Utilizing community‐based data and leveraging local insights can facilitate the tailoring of public health initiatives to fit the specific context of each community, potentially aiding in disease eradication among vulnerable populations (Harvey et al. 2022).

Increasing public awareness about SSPE and measles is crucial, including educating the public about SSPE, its progression, and the importance of vaccination and hygiene practices. To track transmission routes, molecular surveillance can be used, and when coupled with traditional epidemiological data, it provides evidence to support the verification of measles eradication. Sequence data can also differentiate wild‐type infection and vaccination‐related rash. Genotypic monitoring of the MeV is another approach that involves analyzing the nucleotide sequence that encodes the 150 carboxyl‐terminal amino acids of the N protein (N450), an area with significant variability within the genome (Coughlin et al. 2017; World Health Organization 2001). The emphasis should be on the safety and effectiveness of vaccines, advising individuals about early symptoms, and vaccine promotion. Focused awareness initiatives can be implemented in communities with low vaccination rates, concurrently educating healthcare professionals about SSPE and measles. Prioritizing international collaboration is paramount in the collective effort to control measles.

## 7 Treatment and Management of SSPE

The diagnosis of SSPE can be challenging, especially if a patient presents with unusual symptoms. SSPE symptoms can range from severe depression, myoclonus, dementia, and visual impairment to fulminant SSPE, where the progression of the disease is rapid, causing death or the akinetic mute stage within 6 months of onset. The fulminant SSPE can be due to a genetic predisposition; usually, cases of SSPE that have the involvement of pons have a rapid progression and lead to death. Similarly, a case of SSPE presented in a 13‐year‐old girl with HIV showed that SSPE can develop acutely in patients infected with HIV (Vidhale et al. 2021). A few other risk factors have also been associated with fulminant progression of the disease, such as measles infection before 2 years of age, Epstein‐Barr virus, parainfluenza type 1, and impaired T cell‐mediated immunity. Unlike typical SSPE, symptoms caused by fulminant SSPE are abrupt lateralizing neurological signs, seizures, ataxia, and visual manifestations (Karthik et al. 2024; R. K. Garg, Pandey, et al. 2023; Vidhale et al. 2021).

To date, no definitive cure for SSPE has been identified. SSPE results in death in every single case (Memon et al. 2021). Treatments aim to reduce symptoms, hinder disease progression, and prolong survival. The appearance of neurological symptoms such as seizures and gait instability can be treated with anticonvulsants such as clonazepam, sodium valproate, and benzodiazepines (Rocke and Belyayeva 2023; Memon et al. 2021). Antiseizure medications such as carbamazepine (CBZ) are paradoxically effective in the treatment of myoclonus in some patients with SSPE due to their effect on basal ganglia (Samia et al. 2022).

Drug‐resistant epilepsy can be managed with a ketogenic diet that causes inhibition of neuronal hyperexcitability. The ketogenic diet is an anti‐inflammatory diet that is neuroprotective, reduces oxidative stress, improves mitochondrial activity, and suppresses factors that induce apoptosis. Having a ketogenic diet as an adjunctive therapy can be beneficial in symptom control (Samia et al. 2022; Rocke and Belyayeva 2023).

Therapeutic interventions include treatment with corticosteroids and immunomodulating agents. Isoprinosine is an immunomodulator and antiviral that has immunostimulatory effects such as enhancing T cell proliferation and a direct effect on viral RNA levels. Other options, such as amantadine and interferons (IFN), appeared to improve survival outcomes. IFN is used in the treatment of SSPE since it has an inhibitory effect on viral replication, and it has been reported that intraventricular IFN‐α treatment combined with isoprinosine induced remission or stabilization in 44%–55% of SSPE cases (Samia et al. 2022). Although this combination is very effective, it remains out of reach of most patients due to its expensive cost and lack of availability (D. Garg and Sharma 2023). Fusion inhibitor peptides (compound AS‐48), which can combine with the viral fusion proteins, are being developed as future treatment modalities (Memon et al. 2021), and such advancements are imperative to treat a lethal condition like SSPE.

Nucleoside analogs such as Lamivudine and Ribavirin, possessing RNA virus inhibitory properties, have also been used as treatment options. A study has also shown that the oral administration of Ribavirin does not affect SSPE patients, mainly because the concentration required in the CSF to deliver a therapeutic effect was not met (Memon et al. 2021). However, intraventricular infusion of interferon‐ α and ribavirin has shown to be effective in some people (Sonoda et al. 2021). In a case report by Schmitz et al. (2024), remdesivir was used in stage 3 fulminant SSPE. The first two doses of remdesivir appeared to be effective and reduced myoclonic seizures, whereas the third dose was ineffective, and soon after the patient succumbed to their deteriorating condition. It is proposed that the efficacy of remdesivir should also be evaluated, and efforts should be made for earlier treatment onset to limit the neurological damage of SSPE (Schmitz et al. 2024). Lastly, vitamin A supplements effectively reduce the morbidity and mortality of measles infection (Ferren and Mathieu 2019).

## 8 SSPE: Long‐Term Prognosis and Complications

SSPE is a progressive disorder, and early diagnosis is imperative in managing the disease and delaying its onset. Early detection of myoclonus and EEG periodic complexes is important to make such a diagnosis (M. Garg et al. 2022). In some patients, the disease is acute, while in others we see a chronic progression. This progressive deterioration leads to a persistent vegetative state, ultimately causing death (National Institute of Neurological Disorders and Stroke n.d.).

The mortality rate of the disease is around 95%, and the average life span after the initial presentation is 3.8 years, ranging from 45 days to 12 years. The remaining 5% undergo spontaneous improvement, but long‐term remission is infrequent (NCBI Bookshelf n.d.; Nathan et al. 2019). This remission depends upon several factors, including the immune state of individuals and genetic polymorphism (Kocaağa 2022).

Long‐term complications of SSPE include memory loss, dementia, personality and behavioral changes, and visual impairments (Lebon et al. 2021; National Institute of Neurological Disorders and Stroke n.d.). A case report also suggests that doctors should keep SSPE as part of their differential diagnosis for movement disorders. Movement disorders are rare in the early stages of the disease but are common in pediatric SSPE patients (Mondal et al. 2023). Further investigation would allow us to better understand measles‐related complications and improve disease prognosis.

## 9 Future Directions and Research Needs–SSPE

**FIGURE 2:**
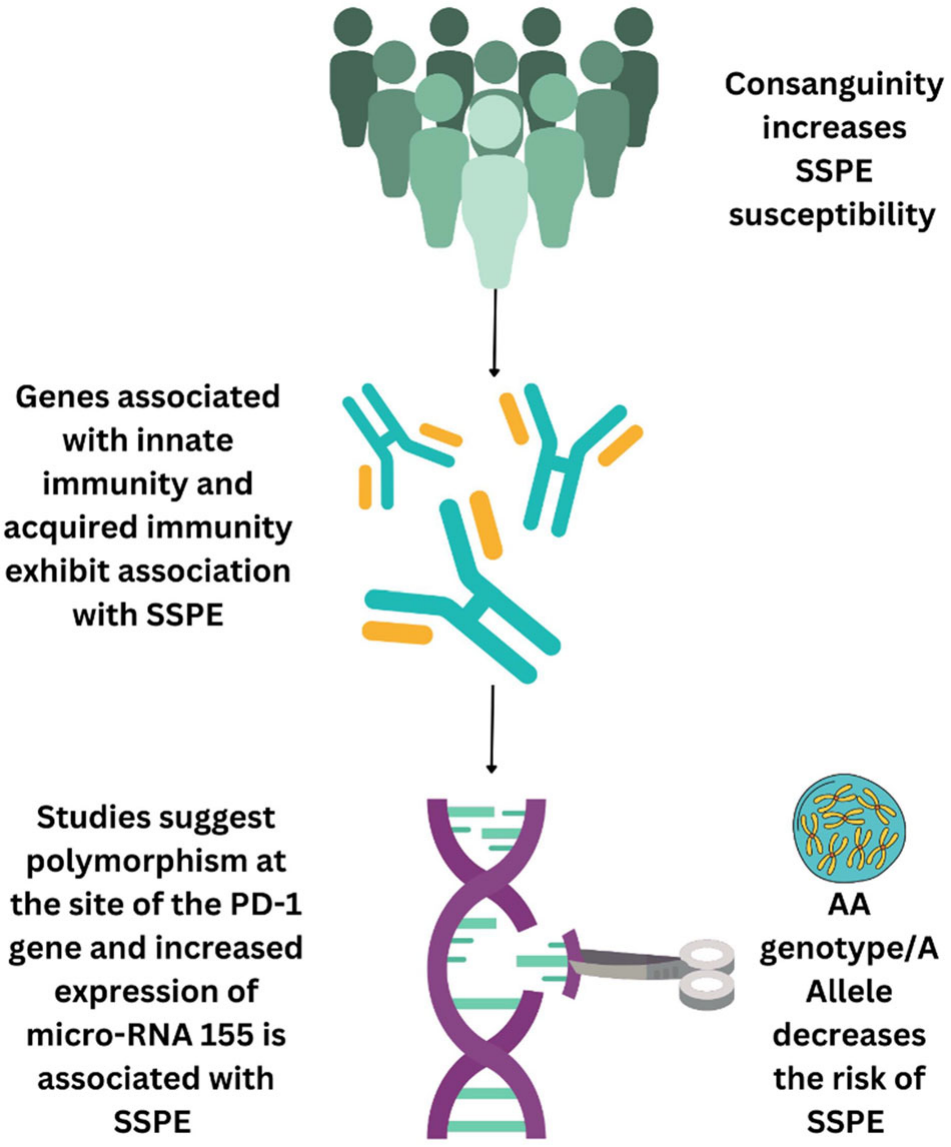
Genetic predisposition of SSPE.

Certain factors increase an individual's vulnerability to SSPE, including consanguinity, specific genetic polymorphism, and immune responses influenced by microRNA. Studies suggest that consanguinity can be a potential predisposing factor for SSPE (Guler, Kucukkoc, and Iscan 2015). Moreover, a report revealed that affected children among the same sibling group, especially twins, suggested increased genetic association (Lebon et al. 2021). Although, for familial forms of SSPE, the genes involved are unknown and require further investigation. Furthermore, it was reported that certain genes relating to innate immunity, including toll‐like receptor (TLR) 3, TLR 4, and MxA, IL28, IL‐29, IL‐17, IL‐18, IL‐12, TNF, and PD‐1, exhibit association with SSPE; however, definitive patterns have not yet been established (Figure 2). (D. Garg and Sharma 2023) Polymorphism at the regulatory site of the PD‐1 gene is also associated with susceptibility to chronic viral infections. Piskin et al. discovered that PD‐1 gene expression in patients with SSPE is greater than in normal individuals. The PD‐1 gene, when binding to its ligand, decreases T‐lymphocyte proliferation and induces programmed T cell death along with suppression of cytokines, hence attenuating the immunity following a measles infection (Piskin et al. 2013).

These findings suggest that a person's genetic makeup can predispose them to acquiring SSPE with a higher risk than the general public. Immune profiling and whole exome sequencing can provide useful insights into host characteristics most influenced by the pathogenic mechanism of SSPE and provide a deeper understanding of the clinical outcomes of SSPE.

Similarly, a study investigated micro‐RNAs and discovered that micro‐RNA 155 expression was greater in SSPE patients. Since there is increased Th2 cytokine synthesis, while on the other hand, there is a decrease in Th1‐specific cytokines, hence the overexpression of micro‐RNA 155 acts as a compensatory mechanism. The same study also reported increased expression of micro‐RNA 181a and 146a. Overexpression of micro‐RNA 181a results in increased T cell hypersensitivity leading to heightened elimination of MeV. Micro‐RNA 146a, on the other hand, decreases MeV reactivation and resultant inflammation (Yiş et al. 2014). Another study uncovers that having the AA genotype or A allele decreases the risk of SSPE, as most healthy individuals expressed this genotype (Figure 2). The AA genotype at the −1188 position decreases the risk of developing SSPE by 2.1‐fold, while the A allele reduces it by 1.6‐fold. However, in this study, there was uncertainty in the IFNG polymorphism as no correlation was appreciated with SSPE patients (Dundar et al. 2016).

In light of this research, further investigation into genetic polymorphism and immune responses of SSPE can bring us closer to discovering new treatment options for SSPE. Recently, medications such as Favipiravir, an anti‐influenza drug, have shown antiviral effects against RNA viruses. A study by Hashimoto et al. (2021) confirmed Favipiravir's antiviral activity against the SSPE virus for the first time by testing its efficacy against the MeV (Edmonston strain) and SSPE virus (Yamagata‐1 strain) in vitro. Favipiravir inhibited viral plaque formation by 50% at concentrations of 108.7 ± 2.0 µM (17.1 ± 0.3 µg/mL) for the Edmonston strain and 38.6 ± 6.0 µM (6.1 ± 0.9 µg/mL) for the Yamagata‐1 strain, like ribavirin (Hashimoto et al. 2021). Keeping these insights in mind, it is imperative to investigate and discover new treatment options providing targeted therapies to patients.

## 10 Conclusion

SSPE is a rare complication of measles, with a life‐threatening prognosis. Immunomodulatory and antiviralagents make its course manageable, but complete eradication remains elusive. Scarce research is available on SSPE due to a limited number of cases. In light of this, our review aims to delve deeper into the disease pathogenesis, clinical implications, and future direction. The best way to avoid the risk of SSPE is by immunization against measles. Bold efforts are required to accelerate the immunization process and curb measles resurgence, especially in developing countries where people are at higher risk of contracting measles. Research indicated that vitamin A supplementation and low glycemic index therapy show promising results in managing the disease. Research on Favipiravir is also underway to evaluate its efficacy against SSPE. This underscores the need for further research and technological advancements to accelerate finding more effective and novel treatment options to manage SSPE.

## Author Contributions

**Zainab Mubbashir**: conceptualization, writing–original draft. **Zoaib Habib Tharwani**: methodology, writing–original draft. **Tilyan Kambar**: writing–original draft, supervision. **Sadia Munawar**: writing–original draft, resources. **Ozem Raphael**: writing–original draft, data curation. **Iman Siddiqui**: writing–original draft, writing–review and editing. **Syeda Ayesha Nadeem**: writing–original draft, writing–review and editing. **Ayesha Amir**: writing–original draft, writing–review and editing. **Amina Ahmed**: writing–original draft, writing–review and editing. **Muhammad Daim Bin Zafar**: writing–original draft, writing–review and editing. **Muhammad Umair Anjum**: writing–original draft, writing–review and editing. **Muhammad Hasanain**: writing–original draft, writing–review and editing. **Abdullah Malikzai**: writing–review and editing.

## Ethics Statement

This study was exempted from the institutional review board's approval because it uses publicly available data that are de‐identified.

## Conflicts of Interest

The authors declare no conflicts of interest.

### Peer Review

The peer review history for this article is available at https://publons.com/publon/10.1002/brb3.70292.

## Data Availability

The dataset supporting the conclusions of this article is included in this article.
